# Unlocking the Next Decade of Proteomics with Standardized, Structured Metadata

**DOI:** 10.1021/acs.jproteome.5c00958

**Published:** 2026-01-21

**Authors:** Tim Van Den Bossche, Ananth Prakash, Tine Claeys, Juan Antonio Vizcaíno, Lennart Martens

**Affiliations:** 1 360916 VIB Center for Medical Biotechnology, VIB, Ghent 9052, Belgium; 2 Department of Biomolecular Medicine, Faculty of Medicine and Health Sciences, 26656 Ghent University, Ghent 9052, Belgium; 3 European Molecular Biology Laboratory-European Bioinformatics Institute (EMBL-EBI), Hinxton, Cambridge CB10 1SD, U.K.; 4 BioOrganic Mass Spectrometry Laboratory (LSMBO), IPHC UMR 7178, University of Strasbourg, CNRS, Strasbourg 67000, France; 5 Infrastructure Nationale de Protéomique ProFI-FR2048, Strasbourg 67087, France

**Keywords:** metadata, SDRF-Proteomics, HUPO-PSI, standards, repositories, funders, software, vendors

## Abstract

The proteomics community has fully embraced data sharing, yet data set metadata provision remains limited, especially at the level of the biological samples and experimental design. This hampers large-scale data reuse, as comprehensive and structured sample context and study design information are often essential for confident, automatic reuse, and (re)­interpretation. Although standards such as Sample and Data Relationship Format for Proteomics (SDRF-Proteomics) and supporting tools are already available, their adoption remains limited. Many researchers lack incentives, and enforcement by journals and repositories remains challenging in practice. Still, metadata defines a data set’s long-term value. We propose a coordinated plan to dramatically improve metadata annotation of publicly disseminated proteomics data. Funders can drive progress by investing in a sustainable, scalable metadata infrastructure. HUPO-PSI plays a central role in setting community standards and enabling validation. ProteomeXchange repositories are key to implementing and supporting metadata adoption. Data producers must treat metadata as a part of their scientific output. Instrument vendors can contribute by enabling the automatic capture of technical metadata. Software developers should embed SDRF-Proteomics metadata into analysis workflows. Finally, journals and reviewers are well positioned to shape expectations and enforce compliance. By aligning efforts across these stakeholders, we can build the road to large-scale, context-aware reuse and unlock the full value of public proteomics data sets.

## Graphical abstract

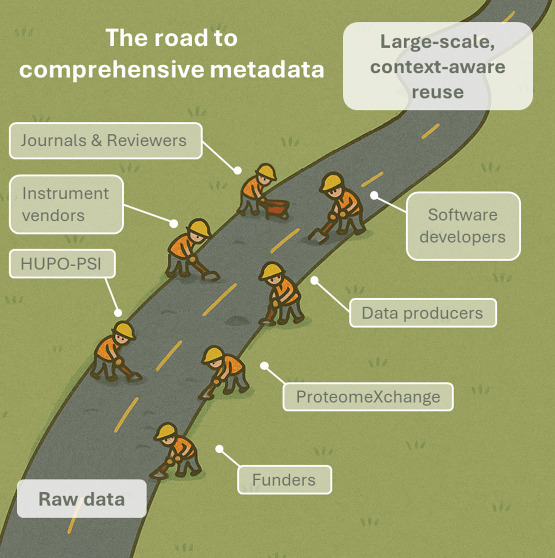

## 1 What Is Structured Metadata, and Why Does It Matter?

The proteomics community has made significant progress in open data sharing. The routine deposition of raw data in public repositories such as PRIDE^1^ and other ProteomeXchange^2^ resources has enabled large-scale data reuse and driven the development of widely used resources and tools. However, this success has not yet fully extended to data set metadata.^3^

Metadata describes the context of a proteomics experiment, from sample collection to data analysis.^4^ It generally falls into three categories: biological metadata (e.g., sample origin), technical metadata (e.g., sample preparation protocols, instrument settings, and data processing parameters), and experimental design (e.g., replicates, fractions, and study variables). This information is essential for the correct interpretation, reproducibility, and reuse of proteomics data.

Even without comprehensive structured metadata, many computational tools and databases have already built upon and demonstrated the huge potential of reusing public proteomics data. Tools such as MS^2^PIP,^5^ DeepLC,^6^ and IM^2^Deep^7^ were trained on public data sets that often lacked complete metadata. Similarly, databases like PeptideAtlas,^8^ Scop3P,^9^ OpenProt,^10^ PaxDB,^11^ ProteomicsDB,^12^ and SysteMHC^13^ routinely extract biological insights from data sets with limited sample annotation. These examples show that reuse is not only already widespread but has also led to tangible, widely used advances in the field.

However, such reuse typically focuses on applications that do not require detailed information about the study design, biological samples, or experimental variables. The absence of structured and complete metadata restricts the types of questions that can be asked and answered. Analyses such as differential protein expression depend on clearly annotated sample groupings and study variables. More broadly, efforts to compare biological conditions, integrate results across studies, or analyze context-specific patterns rely on access to experimental design and sample-level metadata. Without this, large-scale downstream analyses remain fragmented and the full potential of proteomics data reuse cannot be realized.

In many cases, metadata is limited to the highest level only (e.g., sample species and instrument used). Most of the other information provided often appear only in unstructured formats, such as free-text descriptions in manuscripts or supplementary files.^3,14^ These descriptions are difficult to interpret computationally and typically lack the consistency needed for large-scale reuse. This illustrates a key distinction between availability and accessibility: metadata may be available to human readers, but only structured metadata is accessible for automated analysis and integration.

Structured metadata also provide practical benefits. It is far easier to generate a complete methods section from a structured table than to extract relevant metadata from free-text descriptions using large language models (LLMs). Tools such as PeptideShaker^15^ already demonstrate how structured metadata captured during data processing can directly streamline reporting. Based on the parameters provided by the user in SearchGUI,^16^ PeptideShaker can automatically generate a draft of the methods section for manuscripts. This not only reduces the manual effort required to document experimental settings but also ensures consistency and accuracy in reporting, as the metadata are derived directly from the analysis configuration. This asymmetry between generating and recovering metadata resembles thermodynamic entropy: converting structured metadata into a narrative format is relatively straightforward, while reconstructing structured information from free text is far more complex and error-prone, especially at a scale.

To address these limitations, the structured metadata format called Sample and Data Relationship Format for Proteomics (SDRF-Proteomics) was introduced.^4^ Unlike unstructured descriptions buried in manuscripts or supplementary files, SDRF-Proteomics captures metadata in a standardized table-based format that supports automated validation, consistent annotation, and reuse. It covers sample properties, data file attributes, and study variables and is now supported by PRIDE^1^ as the recommended format for structured metadata submissions. By enabling transparent reporting across diverse experimental designs, SDRF-Proteomics reflects a broader community effort to make proteomics data more reusable and interoperable.

To support uptake, several tools have been developed to help researchers create SDRF-Proteomics files. For example, the lesSDRF web tool provides a guided interface for manual annotation.^14^ CUPCAKE (in development, https://cupcake.proteo.info) integrates metadata annotation into LIMS environments through a stepwise wizard. Proteomics software such as MSFragger^17^ or MaxQuant^18^ (from version 2.7.0.0 onward) can now generate SDRF-Proteomics files, which can also be imported into downstream tools like Perseus.^19^ These efforts substantially lower the barrier to adoption by embedding structured metadata capture into existing workflows.

## 2 Why Structured Metadata Submission Remains Elusive

Some consortia, individual laboratories, and annotation initiatives have demonstrated that it is possible to do metadata annotation well. However, these cases remain exceptions rather than the rule, and this scenario is common across the different omics domains.^20^ This situation stems from a combination of practical and structural barriers, which are more commonly encountered in the various omics domains. Below, we outline key reasons that metadata annotation still does not happen at scale.

First, researchers often lack time, clear guidance, or direct incentives to invest in metadata annotation. Academic reward systems prioritize publishing and grant acquisition over the production of well-annotated data sets. As a result, metadata are often treated as an afterthought rather than a core part of scientific output.

Second, metadata is typically evaluated *ad hoc* during peer review, and its presence or quality rarely influences editorial decisions. In most journals, data submission is mandatory, but it is still treated as a technical afterthought, disconnected from the scientific narrative, and often completed solely to meet journal requirements. This marginal role of metadata in the publication workflow reinforces the perception that metadata provision is bureaucratic overhead rather than a core component of scientific transparency and data sharing. As a result, researchers receive little recognition or credit for submitting completely annotated and fully reusable data sets, despite their massively increased long-term value and impact on the community.

Third, even with the availability of tools (e.g., lesSDRF^14^ and CUPCAKE), metadata annotation remains a manual task that requires coordination across multiple actors. Crucial information is frequently spread across the wet lab, omics core facility, and data analysis team. As a result, no single individual holds all necessary information to compile complete and correct data set metadata annotations (e.g., in a SDRF-Proteomics file), making the process cumbersome in practice.

Concretely in the proteomics field, while the above barriers explain why metadata annotation remains uncommon, the problem is compounded by the fact that submission is still largely voluntary. Adoption is slowly increasing in contexts where reuse is anticipated or incentivized. However, researchers receive little recognition or reward for metadata annotation, and most researchers are not aware of its downstream value. Enforcement mechanisms are also lacking. Repositories recommend, but do not require, complete or validated SDRF-Proteomics submissions, and neither journals nor reviewers systematically enforce metadata standards. Without clear incentives or mandates, metadata submission becomes a classic coordination problem: collectively beneficial but individually burdensome.

This raises an important question: what are the consequences of this lack of incentives and enforcement? The absence of consistent metadata annotation severely limits the (large-scale) reusability of many data sets. Crucial experimental context is often lost, making in-depth reanalysis difficult or impossible. As a result, we fail to realize the full return on investment for research that is frequently publicly funded. Without structured metadata, data sharing risks becoming a formality rather than a true catalyst for novel discovery. It undermines large-scale meta-analyses, comparative studies, and the development of new computational methods that depend on well-annotated inputs. In short, insufficient metadata prevents omics data from building effectively on their own output.

## 3 Shared Responsibility: A Call to Action for the Entire Proteomics Community

To make structured metadata the norm, coordinated action is needed across the entire proteomics ecosystem. Indeed, this is not a task that individual researchers can or should be able to bear alone. All stakeholders, including funders, the Human Proteome Organization’s Proteomics Standards Initiative^21^ (HUPO-PSI), data repositories, researchers, journals, reviewers, software developers, and instrument vendors, have a distinct role to play in ensuring that metadata becomes a first-class research output.

### 3.1 Funders: Investing in a Sustainable and Scalable Metadata Infrastructure

Public repositories and metadata standards form the backbone of proteomics data sharing, yet they often operate behind the scenes with limited visibility or recognition. These efforts are rarely considered high profile, but they are absolutely essential to ensure that data remain reusable, interoperable, and trustworthy. Moreover, these repositories have limited funding for “non-glamorous” tasks such as providing support to users, data curation, and other “routine tasks”. There is often the need to balance a heavy submission and dissemination workload with the need to develop user-friendly interfaces and tools, all while staying abreast of new developments in the field. Funders must therefore support both baseline maintenance funding to guarantee long-term operation of repositories and services and also provide spiked funding for development efforts to implement new standards, building submission and validation tools, or integrating metadata across omics domains, among others.

The return on investment is substantial. A study by EMBL-EBI in 2021 ^22^ estimated that the annual operational cost of its 44 open data resources, including PRIDE,^1^ is around £110 million, while their annual use value exceeds £5.5 billion. Although these figures reflect the combined impact of all EMBL-EBI resources and not PRIDE alone, they illustrate the immense multiplier effect of a well-maintained infrastructure. Strategic support from major funding bodies, such as the Wellcome Trust, the NIH, the European Commission, and the Chan Zuckerberg Initiative, is therefore crucial. Sustained investment in standards, validation tools, and long-term repository support may not be glamorous, but it forms the backbone of a functional (meta)­data ecosystem. Indeed, this type of funding does not just keep infrastructure running; it directly and indirectly enables large-scale scientific progress. The community benefits immensely from these resources, and it is up to funders and policymakers to ensure that this foundational layer receives the resources it deserves.

### 3.2 HUPO-PSI: Setting the Standard and Enabling Validation

HUPO-PSI^21^ plays a central role in defining community standards for proteomics, including the SDRF-Proteomics^4^ format. It specifies (meta)­data requirements and relies heavily on controlled vocabularies (CVs), including the PSI-MS CV maintained by HUPO-PSI.^23^ These CVs ensure consistent terminology for instrument metadata, sample descriptors, and experimental protocols and are accessible through resources such as the Ontology Lookup Service (OLS). HUPO-PSI also ensures that standards evolve in line with the needs of the field (e.g., with the advent of novel instruments or specific applications such as metaproteomics and single-cell proteomics, for which such guidelines should be developed in collaboration with community initiatives like the Metaproteomics Initiative^24,25^ or the HUPO Single-Cell Proteomics Initiative, respectively). HUPO-PSI further codevelops semantic validation specifications, as implemented in the SDRF-validator. This validator checks whether metadata files contain the required fields and uses appropriate ontology terms in these fields.

A key strength of a well-designed validation system is its ability to derive rules directly from the written standard, ideally enabling configuration without extensive programming. While this is not yet implemented for SDRF-Proteomics, it is a goal the community should work toward. This approach enables researchers lacking programming experience to contribute directly to this project. To support effective adoption, the validator must not only detect issues but also provide clear, actionable error messages. These messages should guide users in correcting mistakes without requiring deep technical knowledge. To ensure transparency and long-term sustainability, these rules should be revised, openly documented, and updated regularly as the community needs to evolve. HUPO-PSI also plays a key role in raising awareness of metadata standards and providing training materials that explain how these support reproducibility, transparency, and reuse in proteomics.

### 3.3 ProteomeXchange Repositories: Implementing and Supporting Metadata Adoption

ProteomeXchange^2^ is the central coordination framework for public proteomics data repositories such as PRIDE.^1^ As a consortium, it should play a key role in ensuring that structured metadata is not only collected but also stored and made accessible for reuse. This requires repositories to adopt shared metadata formats such as SDRF-Proteomics^4^ and to implement consistent, user-oriented submission workflows. Equally important is the storage of metadata in structured, queryable formats that enable automated reuse, integration with other omics data, and large-scale meta-analyses.

Repositories must provide both user-friendly submission interfaces and programmatic access through APIs, lowering barriers for both data depositors and reusers. PRIDE, for example, has taken concrete steps by supporting the inclusion of SDRF-Proteomics files during submission and offering integrated feedback mechanisms for metadata quality control. While the submission of SDRF-Proteomics files is currently optional yet recommended, it demonstrates a clear direction toward improved metadata practices. Continued improvement of these tools along with clear documentation and practical guidance will be essential to support broader adoption. In parallel, all ProteomeXchange partners should commit to the long-term maintenance and evolution of metadata standards, ensuring that structured metadata becomes a robust and reusable foundation for proteomics research. Once the infrastructure, tools, and community support are in place, the submission of structured metadata files should become a mandatory part of public data deposition. Importantly, it should be reiterated here that this onus cannot be put on repositories without commensurate investment by the community through its funders.

Moreover, note that we have not even touched upon the added complexities introduced by the sharing of clinical and pathogen-related proteomics data, for which a plethora of additional safeguards and corresponding development is necessary.

### 3.4 Data Producers: Treating Metadata as Part of the Scientific Output

Individual researchers, research groups, and consortia should treat metadata as an integral part of their research output. Too often, it is seen as an administrative burden to complete after the study is finished, yet data sets often outlive their publications in terms of relevance and reuse potential. Providing structured metadata ensures that data sets can be interpreted, reused, and integrated into future studies, both by others and by the original authors themselves.

Creating SDRF-Proteomics files should become standard practice, ideally while the study is ongoing rather than at the submission stage. Thanks to tools like lesSDRF,^14^ CUPCAKE, and exporters integrated into MaxQuant^18^ and MSFragger,^17^ this ongoing annotation process is becoming easier and more accessible. As the SDRF-Proteomics ecosystem continues to evolve, metadata submission will become less time-consuming and more directly beneficial, providing a crucial element of a functional and open data exchange ecosystem in proteomics.^26^

### 3.5 Instrument Vendors: Enabling Automatic Capture of Technical Metadata

Instrument vendors play a key role in ensuring that technical metadata can be captured consistently and at scale. By supporting automated export of acquisition settings, sample queue information, and instrument parameters in the SDRF-Proteomics format, they can reduce the workload for researchers and improve metadata completeness. Better yet, the ability to design experiments in the SDRF-Proteomics format and upload this design directly into the instrument software could streamline both sample acquisition and metadata capture. For example, researchers could define sample names, biological conditions, technical replicates, fractionation steps, and file naming conventions in advance using an SDRF-Proteomics spreadsheet. Instrument software would then read this file to automatically populate the acquisition queue and apply the correct labels and annotations to each run. This approach would ensure that each raw data file is automatically and accurately linked to its associated metadata without requiring additional manual input. As a result, metadata capture becomes embedded at the beginning of the experimental workflow rather than added retroactively, reducing human error and promoting structured submission practices.

### 3.6 Software Developers: Embedding SDRF-Proteomics Metadata in the Data Analysis Workflow

Both academic and commercial software developers can further facilitate metadata capture by integrating the SDRF-Proteomics support directly into data processing pipelines. This includes both reading SDRF-Proteomics files to interpret experimental design (e.g., fractions, conditions, and replicates) and writing output files that retain metadata annotations throughout downstream analyses. Tools such as MSstats^27^ have already demonstrated how capturing metadata from the input side enables automated, reproducible analysis. Exporting metadata directly from software also reduces the chance of human error, ensuring a higher consistency and completeness.

As a side note, electronic lab notebooks (ELNs) are sometimes proposed as a solution to metadata capture, but in practice, these rarely offer a reliable or scalable option. Many ELNs are used in a *post hoc* manner (researchers fill them in after experiments have been run), and there is little consistency in how these are used across different laboratories or institutions. Moreover, ELNs are often isolated systems, making it difficult to export metadata in structured, standardized formats suitable for reuse or integration.

In contrast, when analysis software supports reading from and writing to SDRF-Proteomics files, it becomes possible to ensure consistency across the entire data lifecycle. Metadata defined before the experiment can guide the experimental setup and acquisition; metadata captured during data processing can be written back to the same SDRF-Proteomics file. This ensures that sample and file annotations remain aligned throughout the workflow, enabling reproducibility, interoperability with downstream tools, and compliance with community standards. Rather than treating metadata as a final reporting step, this approach embeds it as a living component of the experiment–analysis cycle.

### 3.7 Journals and Reviewers: Shaping Expectations and Enforcing Compliance

Journals hold a uniquely powerful position in academic publishing. Their policies have been instrumental in making data deposition a standard requirement in proteomics, largely by mandating submission to public repositories. A similar shift is now needed for metadata. High-impact journals in particular must take the lead in requiring structured metadata submission, such as SDRF-Proteomics files, and in coordinating expectations across the proteomics community.

To support this transition, journals should provide clear guidance to both authors and reviewers on metadata expectations. These should align with HUPO-PSI standards and integrate seamlessly with existing submission workflows that already require ProteomeXchange accession numbers. Journals should not require direct metadata submission to the journal system but instead ensure that metadata is properly deposited in ProteomeXchange repositories. Editorial policies should be clearly communicated and consistently enforced to avoid ambiguity.

Reviewers played a key role in ensuring compliance. Just as they assess data availability, they should evaluate whether structured metadata is present and sufficiently detailed to support reproducibility. When metadata is missing or incomplete, clear journal policies should encourage reviewers to request improvements prior to publication.

## 4 Recognizing the Value of Metadata

While the provision of structured metadata requires some additional time and effort from the data generators, it should not be seen as an administrative burden. It is a scientific necessity that underpins transparency, reproducibility, and reuse in proteomics. Metadata is what allows today’s data sets to serve as the foundation for tomorrow’s discoveries. It supports the development of AI-driven tools and creates opportunities for large-scale analyses that go far beyond the scope of any individual study.

The benefits of structured metadata are both collective and personal. By annotating a data set, researchers contribute to a shared resource that can be explored, reused, and extended by others. In turn, they gain access to an expanding pool of well-annotated data sets from across the field, which they can reuse in their future research. The return on investment is therefore exponential. Importantly, high-quality metadata not only benefits others; it also benefits the original data producers by improving visibility, discoverability, and citation of their work.

The field of proteomics has been a pioneer in global structured data sharing. This culture must now be extended to the metadata. The necessary standards, tools, and infrastructure are already in place or under active development. What is needed now is coordinated action across the entire ecosystem. Funders must provide both long-term maintenance and spiked development support for these shared community resources. Researchers must treat metadata as part of their scientific output. Journals must set clear policies. Repositories must be provided with the means to implement and maintain robust metadata support. Instrument vendors and software developers must enable the automatic capture and propagation of metadata throughout the experimental and analytical workflow.

The community has already successfully implemented such a vision for raw and processed data; now is the time to do the same for metadata. Given the right support, the proteomics community can ensure that its data is not only openly available but also richly annotated and truly reusable for maximal benefit to all!
